# A Privileged Medical Class

**Published:** 1913-04

**Authors:** 


### A Privileged Medical Class.

After Them Again.—Like an avenging Nemesis, Dr. G. Frank Lydston is camping on the trail of Simmons and the Octopus, showing up more of their rascality. In a neat pamphlet just issued, Dr. Lydston charges that the management of that great trust are above the law, that they are a privileged class ("the king can do no wrong"), and that the Federal authorities wink at their arrogance. In particular, he charges that in the matter of appointments on the reserve medical corps of the army those who are recommended by this great usurper of legislative functions and dictator of all that pertains to medicine in this free country, are commissioned by the War Department, without examination, and that of those who have been so commissioned, "the first batch comprised the editor-manager-boss of the A. M. A. and twelve of his official family. Every local official of the A. M. A. is now in the Medical Reserve Corps Association of Chicago." Dr. Lydston charges that the medical department of the army is dominated by, and subverted to the base uses of this "dominant ring of medical politicians," that the surgeon general is working in conjunction with this ring and wishes these privileges to continue (appointment without examination, and the privilege of private practice, without examination and license), to be unlimited and unfair to the profession at large.

These are grave charges. Dr. Lydston says he stands prepared to prove them. The matter certainly demands the attention of the general of the army, and if such conditions exist, President Wilson should make some removals in the medical department.

UP AGAINST IT.—Apropos: It seems that the existence of a great medical trust, a monopoly, a usurpation of authority, a conspiracy in restraint of trade (?) has been recognized, and some one has arisen, bold enough to strike, and test it before the courts. The Octopus assumes to regulate medical education and say which colleges may teach and which may not, as well as to regulate the manufacture of pharmacals, and to say which medical journals may exist and which may not, and we of the medical press are too weak to kick. But here comes a medical college that has the power to hit back. I reproduce the following from the *Chicago Tribune* of March 3:

PREDICTS JAIL TERMS FOR "MEDICAL TRUST" MEMBERS.

Charles J. O'Connor, attorney for Jenner Medical College, who on Friday started suit for $500,000 against the American Medical Association, Dr. Arthur D. Bevan, and ten other physicians, has written an open letter to Dr. Bevan, in which he intimates that the doctor and his associates are in danger of jail for violating the anti-trust law.

"Our institution was never examined by any one representing you and your so-called council of medical education but one," he says. "That was by Dr. N. P. Colwell, who frankly stated his surprise at finding the institution in such excellent condition.

"The unfortunate part is that he did not report to you and men of your standing. Presumptively he reported to Dr. Simmons, secretary of the American Medical Association, whose record is so bad and so dark that it has been assailed time and again, openly, notoriously and publicly, by many men of as great eminence even as yourself in the profession, one of whom is Dr. G. Frank Lydston, of whose specific charges against Dr. Simmons you are undoubtedly aware.

"The National Cash Register Company of Dayton, Ohio, operated exactly along the lines as you are operating in your efforts to crush competition."

